# Glycosuria

**Published:** 1882-12

**Authors:** Oscar C. DeWolf

**Affiliations:** Professor of State Medicine and Public Hygiene in Chicago Medical College


GLYCOSURIA. By OSCAR C. DEWOLF, A.M., M.D., Professor of State Medicine and Public Hygiene in Chicago Medical College.

It requires but little reflection etymologically, to justify a preference for the word glycosuria in naming the disease upon which Aretæus first wrote as diabetes, in ignorance of the saccharine and non-saccharine conditions existing in what are now known to be widely different diseases. Though diabetes mellitus has been used to designate the constant, and glycosuria the temporary occurrence of sugar in the urine, the absurdity of any such distinction is too apparent. It is safe to predict that the terms chronic and temporary glycosuria will take the place of the older but inexact names mellituria, etc. *Zuckerharnruhr* is an expressive German equivalent.

The literature of the subject is in a scattered and generally inconclusive state, though this is not peculiar to articles upon any one disease. The best work accomplished in an investigation of glycosuria is that by the late Claude Bernard.* With this physiologist's conclusions (familiar to all as the glycogen and glucose theory) as a *datum plane*, to use an engineering term, thousands of "contributions," good, bad, and useless, have been presented to the medical reader. A glance at the seven pages of bibliography by Senator,† and through the pages of the American *Index Medicus*, suffices to discourage any other than the sciolistic student, whose sole object is to *appear* to know. One of the *Esse Quam Videri* genus would soon learn that the literature of the

* Claude Bernard, Mémoires de la Soc. de Biologie, 1849, vol. i., p 221. Nouvelles Fonctions du Foie Considérée comme l' Organe Producteur de Matière Sucrée. Paris, 1853.

† Senator, Ziems en's Cyclopædia, vol. xvi., pp. 851—858.

subject is immensely more voluminous than Senator indicates, and that between compassing it and indulging in original experimentation and investigation an ordinary life-time would not suffice. Doubtless nine tenths of what has been written upon diabetes mellitus is worthless, owing to lack of proper education or surroundings on the part of the writer, or insufficient time to properly analyze cases. The earnest student cannot reform the world; he must take things as they are, and make the best of them. In this endeavor he will be straightway appalled at the polemics indulged in by "authorities," and upon points he imagined were fundamentally established. T. Lauder Brunton* gives a fair review of this aspect of the mellituria question, with the discussions, denials, and controversies between contemporary and other authors and investigators, such as Rollo, Tiedemann, Gmelin, Ambrosiani, Dumas, Bouchardat, Liebig, Bernard, Bouley, Poggiale, Longet, Pavy, Flint, and others. The labors of the ideal physician are presumed to be largely altruistic, but I cannot avoid the conviction that were some such brilliant individuals as Newton, Descartes, Hunter, Huxley, or Tyndall, afflicted with thorough medical educations and glycosuria at the same time (providing the latter impaired their minds and usefulness as little as the former), their egoistic efforts might and in all probability would result in something definite in this direction.

In the midst of distracting public duties the subject of glycosuria has recently occasioned me much concern, and I have attempted its review, the results of which I submit in the shape of conclusions rather than disquisitions and re-presentations of the treatment in text-books accessible to all; not in the spirit of Tom Hood's "Otaheitan cook, who thought no food was fit to eat till he had chewed it," but rather with the *animus* of Chambers, who recognized the impossibility of treating any disease (scientifically) without having a hypothesis as to its nature, and as to the best means of combatting the disorder. Later in the year I hope to be able to communicate to this Journal the result of some clinical observations in the matter of glycosuric treatment.

Chemical text-books, and works on the practice of medicine discuss far more fully than could be done in this essay such matters

* T. Lauder Brunton, Reynold's System of Medicine, vol. iii., p. 35, et seq.

as urine-analysis and the relative quantities of fluid and food ingestion and excretion. In fact, so much space in current print is allotted to a consideration of these threadbare points that only this mention will be made as an excuse for omitting further reference to them.

Naturally enough the disease was in ancient times supposed to be seated in the kidneys. Every tyro in medicine now knows that to Claude Bernard we are indebted for the discovery of the connection between glycosuria and a disarrangement of the liver function. That puncture of a portion of the lower floor of the fourth'ventricle will produce sugar in the urine is also well known, better known than that diabetes insipidus has a so-called center in the medulla just above the glycosuric point. Still less known is it that lesions of the sympathetic system below the ventricular point may likewise cause mellituria, and that pathological conditions of the vagi may induce phases of the disorder.*

Glancing over the various accounts of diabetic ætiology, we find that heredity as a predisposing cause, or a factor at all, is debatable. Granting that neuropathic parents have had offspring in whom glycosuria has been developed, the question of heredity then narrows itself to the inquiry whether defects of the nervous organization may not assune different ways of manifestation in offspring just as cachexia does. It is agreed that men are more liable to glycosuria than women, and that it may appear at any age; but until we have more pains-taking statisticians scattered over countries other than those reported from by Griesinger, Seegen, Schmitz, and Dickenson, we may conclude that we have imperfect data from which to make deductions. Trousseau regards corpulence as a predisposing cause, and when we consider the chemical relationship of fat and sugar it is not to be wondered at that retrograde metamorphosis would be as liable to occur as that sugar should form fat by oxidation. Thus obesity might be considered ætiological as well as predisposing. Another view would be that starch or glycogen, instead of undergoing transformation into fat directly or indirectly, by some perversion of function, became glucose instead. So far as I am aware these probabilities have had no consideration anywhere.

* Marcus and Wiet, Le Progrès Médical, No. 21. Proc. Soc. Biol., May, 1881.

Reviewing the neurologial aspect of the disease, there seems to be very much yet to be done in this field. Cyon and Aladoff* figure the course of the vaso-motor nerves of the liver from the base of the brain downward. This can but be hypothetical. Kronecker,† at Leipsic, confines all vaso-motor centers to the floor of the fourth ventricle, but Luys and Jewell‡ suppose that there is a continuous column of such centers for the whole body in the medulla and cord, while Brown-Séquard extends them to cerebellum and brain, and Clevenger|| agrees with the latter, but goes further in extending them to the entire cerebro-spinal system. Cyon and Aladoff give the glycosuric tract as passing down the spinal cord to some of the communicating branches of the sympathetic, thence through the splanchnic nerves to the liver. Admitting that some such tract undoubtelly exists, the next step is to ascertian how a lesion would operate through it to produce sugar in the liver. The generally accepted view is (from some very notable experiments) that dilatation of the hepatic blood-vessels through vaso-motor paralysis, thus induced, produces engorgement, and that sugar in the urine follows. As to *why* sugar-production follows this interference with liver function much has been written, and doubtless much more will be. Glycosuria has been likewise produced by injuries of the cerebral lobes, cerebellum, optic thalami, and sciatic nerve, according to Schiff, Eckhard, and Pavy, which fact would invalidate the idea of a single tract and the widely noticed ventricular irritation of Bernard as a cause, and account for glucose being somewhat commonly found in the urine of those suffering from any mental or nervous ailment.

Taking these things into consideration, we may well wonder whether the transfer of the disease from the kidneys to the liver as a seat is more than a step toward a clearer, broader view of both the disease and bodily functions in general. Brunton thinks "we are justified in believing that the sugar which is present in the blood, becomes converted by the aid of a ferment in the blood muscles, and probably lungs, also, into lactic acid and glycerine,

---

* Cyon and Aladoff, Bulletin de l'Acad. de Petersbourg, vol. viii.

† Vulpian, Rev. Scient., 1874, No. 35.

‡ Journal of Nervous and Mental Disease, vol. 1., p. 116.

|| Op. cit., October, 1880.

and then undergoes combustion, thus sustaining the temperature of the body. Supposing, however, that this ferment is deficient, a greater or less proportion of the sugar will not undergo conversion into acid, and will then remain unconsumed." The temptation to be discursive and polemical at this point is very great but I prefer to present deductions which I have made while referring to some recent articles bearing more or less directly upon the neurology of the subject.* Bernard himself enlarged upon his original views, and in a communication to the Academy of Sciences January 10, 1876, reported in the *Revue Scientifique*, summarizes the principal points elicited, which seem to have been overlooked by many who have written upon glycosuria. It affords a good *résumé* of our present knowledge of the subject and is worth reproducing here:

"Animal blood always contains sugar. This glycæmia depends on a nornal function of the liver. These facts were established by Bernard and those succeeding him. The physiological production of sugar in the liver is subject to the influence of the nervous system, and by wounding a particular point in the fourth ventricle not far from the origin of the vagi nerves, the sugar is developed superabundantly, rendering the animal rapidly diabetic; from this arose the theory of the glycogenic function of the liver. This function only becomes developed at a certain period of intra-uterine life, but sugar is none the less lacking in the organism undergoing evolution. Sugar exists in the allantoid and amniotic liquids and in the urine, and diabetes is, after a fashion, the normal condition of the fœtus. Bernard from this regards glycogenesis as a general physiological phenomenon, *accompanying all the manifestations of life in animals and plants.* Later, the discovery of the glycogenic matter to some extent changes the face

---

* Painful Symmetrical Neuralgia in Diabetes, Sciatic and Dental. *Le Progrès Médical.* M. J Worms, Session Paris acad. Med., September 10, 1880. Lesions of the Brain in Diabetes, Stevens' Triennial Prize, January 1, 1882 (to be awarded March, 1882, at the anual commencement of the New York College of Physicians and Surgeons). Changes of the Sympathetic in Diabetes, Poniklo. *London Lancet*, May, 1878. Influence of the Nervous System on the Formation of Sugar, M. Laffont, *Progrès Médical*, No. 10, 1880. Glucose Tests, *Science* (New York), December 24, 1881, p. 616. The Dynamo-Chemical Theory of Diabetic Glycæmia, M. Fleury, Acad. de Médicine April 10, 1877, Bull. Gen. de Therapeutique. The Teeth in Diabetes, Magitot, *Journal de Médecine de Bordeaux*, January 1, 1882. Clinical Observations of Diabetes Mellitus, New York Academy of Medicine, stated meeting, February 16, 1882 *New York Medical Record.* February 25, 1882.

of the problem in connecting it with one of the most difficult questions of general physiology, that of the intimate nutrition of the tissues. The theories of nutrition have always allotted to the blood the principal part in the chemical changes that take place in the living organism, but M. Bernard has shown that instead of seeking directly in the blood for the substance preceding the sugar and producing it, it is necessary to place it in the hepatic tissue itself."

M. Lancereaux, it seems to me, took a step in the right direction, in announcing two diabetic types, in a paper read before the French Association for the Advancement of Science, August, 1879. Lancereaux claims that there are *fat and lean types*. The first begins insidiously between the ages of twenty and thirty with obesity, death occuring from phlegmon, rarely from pulmonary consumption. The lean cases spring suddenly from a condition of previous good health, and last from one to six years, or more frequently terminate with phthisis pulmonalis which begins with chronic pneumonia. *Destruction of the pancreas* was recognized in two of the "lean" cases examined, and he infers a direct relation between pancreatic destruction and glycosuria, claiming that there are good grounds for a nosological distinction of these two varieties.

While the obese cases may be simply such as Senator considers predisposed, it would be well tos separate all such cases hereafter and see whether there was the uniformity of phenomena mentionen by Lancereaux. Similarly, I fancy, we may be enabled to diagnose the especial lesion and its seat by some such sensible proceeding, instead of contenting ourselves with the bare fact of sugar being constantly found in the urine. A rational system of treatment can thus be inaugurated which will enable us to address ourselves intelligently to *causes* instead of effects. For example, neuropathic cases might find especial benefit from neurotics, while those originating in obesity would derive no advantage from such treatment. All measures adopted in the latter cases should partake of an effort to aid in decreasing fat production and an interesting point in this connection is afforded us by Immermann :*

---

* Immermann. Corpulence. Ziemssen's Cyclopoædia, xvi., page 647.

"In violent voluntary motions of the body not only will less fat be produced, but, owing to the more energetic process of oxidation in the tissues more ipogenous material will be destroyed." Hence the observations of Bouchardat, Kuelz, and Trousseau, that active muscular movements are capable of lowering the excretions of sugar, have a rational foundation in the presumption that both these hydrocarbonaceous substances, fat and sugar, are burned up in the muscles under proper conditions, and that both are fuels (so to speak) which if allowed to accumulate become pathological and consequently pernicious. On the other hand, the inappropriateness of much exercise for the enfeebled non-obese cases might soon be made apparent by trial.

Although glycosuria does not frequently fall to the care of general practitioners, it has befallen me to have charge of several cases directly and indirectly, naturally awakening a solicitude not to be appeased by our inchoate literature of the subject.

The fact that one half of these cases undoubtedly originated in neuroses of a mental type could not fail to impress me with the conviction that the diabetic center in the floor of the fourth ventricle might have become involved by either an extension of a lesion originating above this point or by direct congestion thereabouts. The patients mentioned are engaged in large manufacturing and mercantile enterprises, and uniformly their glycosuric traubles began after having been subjected to great mental strains. This set me to overhauling the subject, with the results which I hesitatingly submit in condensed from in the shape of conclusions at the end of this article.

Prout was first in calling attention to the phase of glycosuria known as diabetic coma. The later workers in this field (Petters, Sanders, Hamilton, Kussmaul) somewhat uniformly overlook the neurological aspect of the dyspnœa which ushers in the coma. It is very evident that a nerve degeneration which could cause the interference with the liver functions, and thus induce diabetes could as well extend to the pneumogastric centers or fibers, and thus produce the respiratory insufficiency directly through failure of innervation.

Petters urged the acetone poison theory as a cause of the coma, but Sanders and Hamilton* satisfy us that acetonæmia may exist without the coma. It is sufficiently proven that the blood in these coma cases does evolve acetone, but that it is a factor in the production of dyspnœa seems to have been disproven. Ruppstein† gives an equation illustrating the formation of acetone, alcohol, and sodium hydrogen carbonate from sodium ethyl diacetate. In this decomposition the latter absorbs two molecules of water, which fact is again indicative of the abstraction of the blood serum alluded to in another part of this paper as productive of polydipsia.

Quincke‡ and Steel, cited by Gamgee|| mention two cases where the coma was transitory, which would disparRge the neurological view and lead us to search for a chemical or at least less organic cause.

Something in this direction, not alluded to by others, occurred to me when considering the power possessed by animals to deoxidize saccharine substances in fat conversion. By taking a certain number of molecules of carbonic acid water, and oxygen from glucose, the elements of the ordinary forms of fat are furnished: $20C_6H_{12}O_6$ (sugar) $= 2C_{57}H_{110}O_6$ (stearin) $+ 6CO_2 + 10H_2O + 43O_2$. Quincke, who rejects the acetonæmia theory concludes that the comatose condition is due to a toxic action and not to a suddenly developed nervous lesion .Gamgee calls attention to the remarkably intense fatty infiltration of the liver in two cases in the Royal Infirmary. Hoppe Syler § states that the proportion of fat increasad greatly in the blood in four cases of diabetes.

Notwithstanding the note that in one of the Infirmary cases the blood did not seem to be particularly venous, I cannot help believing that the liberation of such great quanitities of carbonic anhydride in fat formation was a direct cause of the coma. At least the view is submitted to physiological chemists as worthy of attention. Then, again, the observation of Foster¶ in support of

* Gamgee, Physiological Chemistry of the Animal Body, vol. i., page 169.

† Centralblatt, 1874, No. 55

‡ Ueber Coma diabeticum, Berliner Klinische Wochenschrift, 1880, No. 1.

|| Op. cit.

§ Physiologische Chemie, page 482.

¶ Diabetic Coma Acetonæmia, British Medic l Journal. 1878, vol. i., p. 78.

the acetone theory were instrumental in establishing the lipæmic condition of the blood in diabetes, and Czerny's account of symptoms in a case of fat embolism led Sanders and Hamilton to advance the theory that "the peculiar terminal dyspnœa and coma of diabetes are due to lipæmia and fat embolism rather than to acetonæmia. Setting aside the acetonæmia theory of diabetic coma as untenable, but being forced to acknowledge that the neuropathic and chemical explanations suffice in some cases, it would seem to justify the view that this diabetic terminal disorder had a multiple origin. In some cases we may conceive that the direct cause is the $CO_2$ poisoning the blood in lipæmia, a view warranted by the fatty infiltration of the liver found in the cases cited by Gamgee; that fat embolism from lipæmia may also be a cause in one class of cases, while pneumogastric nervous interference mainly by progressive degeneration either of centers or nerve tracts may account for another series.

A recent contribution against the acetonic idea, the preliminary communication by Dr. Rudolf von Jacksch,* may be cited as embodying the following conclusions regarding acetonuria in fever generally: First, a red color is produced in the urine by the addition of chloride of iron not only in diabetic coma, but during the course of diabetes without coma, and with great regularity in the eruptive stage of some acute exanthemata. Next come Siebold and Bradbury,† who state that instead of acetone it is aceto-acetic ether that is found in diabetic urine, and setting forth the statements of Tichborne and Attfield, it seems by no means established that the chemists are certain of their tests for either the one or the other of these alleged glycosuric accompaniments.

Lubimoff‡ found "the inferior ganglion of the vagus-trunk atrophied" and abnormally rich in pigment," and Henrat§ once observed a tumor of the right vagus at the level of the root of the lung." Though in neither of these cases is there mention of coma, it is worth while to admit the strong probability of this aspect of

---

* Prager Medicinische Wochenschrifta, October 5, 1881.

† Martin's Chemists and Druggists' Bulletin, January, 1882.

‡ Virchow's Archiv, vol., lxi. p. 145.

§ Bull. de la Soc. Méd. de Reims, 1874, No. 13 (cited by Senator).

the disease not to be due to any single cause in all cases, but often to separate or conjoint causes, among which in my opinion, the $CO_2$ poisoing may rank first, fat embolism second, and pneumogastric failure third. On the matter of fat and glycogen-production from sugar and starch in the animal body an interesting chapter may be found in Foster's Physiology, recently published.*

Before dismissing the consideration of fatty infiltration it would be well to call attention to the statements of Beal, Frerichs, and others, that hepatic fat diminution is the rule in glycosuria, and that infiltration occurs in the corpulent, while fatty degeneration of the liver may occur as the result of cachexia and pulmonary phthisis.

As to the cause of the polydipsia, a view that at one time I regarded as original with myself alone, but which I find has been already urged, seems to satisfy the conditions, but the idea has not been properly worked out heretofore:

Setting all theory aside, the simple fact remains that there is an excess of sugar produced in the system in this disease; it is also undeniable that this sugar has an amylaceous origin. Now, if we take the chemical formulæ of starch and sugar, it is obvious that in the conversion of the one into the other *water is abstracted* from the system. Thus $C_{12}H_{20}O_{10}$ (starch) $2C_6H_{12}O_6H_2O$ (starch sugar) differ by $4H_2O$, and in the formation of two molecules of dextrose (Kekulé's term for starch sugar, Miller suggesting the word glucose as generic) from one molecule of starch four molecules of water disappear. The great thirst in cholera is known to be due to the loss of the watery constituents of the blood, and the diabetic thirst may be similary caused. That the great amount of fluid ingested in the latter disease should fail to assuage thirst is not to be wondered at when we consider that the sugar production goes on comparatively at a distance from the intestine in viscera and vessels not readily accesible or to be rapidly reached by draughts of water intended to resupply the abstracted serum. This process may be compared to the attempt to extinguish a fire in some inner room of a house by deluging the building and all the other apartments with water.

---

* Foster's Physiology, edited by Reichert, American edition, 1880, p. 590, et seq.

The new science of organogeny promises to give us clearer ideas of hepatic functions as well as broader views of man's position in the animal scale. Heretofore we have been compelled to rest content with the meager details set forth so voluminously by Richard Owen, but upon which Huxley improved immensely. Gegenbauer inaugurated with Hæckel an era in these matters, and to-day the apperance of Balfour's second volume on embryology, devoted more especially to vertebrates, is hailed by biological and pathological students everywhere with satisfaction.

Comparative cellular pathology and histology generally, in connection with a less narrow physiological chemistry, will achieve wonders in the future toward a better understanding of the life processes.

When we consider how much vagueness there is in the writings of those who have sought an explanation of the office of the liver, as well as of other structures in the animal economy, a vagueness prevailing in the works of such as Claude Bernard and Frerichs, is it not time to think that want of catholicity is at the bottom of our ignorance? Of course it is not to be expected that one or a score of investigators can cover a universe of comparative histological observations, but it is safe to assert that until organogeny has been thoroughly worked out in other vertebrate forms than man we are doomed to narrow-minded views of the significance of important viscera.

CONCLUSIONS.

Glycosuria is a more appropriate name for the disease known by the several designations diabetes mellitus, mellituria, etc., though glycæmia is the true pathological condition.

The literature is more voluminous than satisfactory, and consists mainly of repetitions of the older views.

Too many changes have been rung upon the necessity for abstinence from amylaceous diet, urine analysis, etc., and too little has been accomplished in the way of autopsies or a consideration of the probability of rational chemical treatment.

Statistics are lacking in establishing heredity and other matters as factors. Climatic influences do not seem to have been considered at all, and especially have the neurological bearings of the disease received too little attention except in the routine of "diabetic vaso-motor tracts."

Similarly the liability to the disease of sexes and at different ages is only apparently settled.

Too many uneliminated sources of error have been mixed up with the records as far as I have examined them.

The causes of glycosuria are multiple, hence the necessity for individualization in diagnosis and treatment.

The Bernardian view that "glycogenesis is a normal physiological phenomenon accompanying all the manifestations of life in animals and plants," deserves serious consideration in connection with accordant modern biological revelations. The tendency has been to consider the disease from the pathological stand-point alone. Nevertheless Bernard does not seem to have laid any stress upon the liver being simply the sugar-forming organ *par excellence* only, and that every organ, in fact every cell, possesses this function to a greater or less extent.

Comparative histology and physiological chemistry promise to become powerful aids in clearing up obscure points in disease, and inasmuch as we are to consider glycæmia from the physiological rather than the pathological stand-point, especially should the recent science of organogeny be cultivated.—*Boston Medical and Surgical Journal.*

